# Triple-bolus CT urography: an optimized approach for vascular assessment in ureteropelvic junction obstruction

**DOI:** 10.1093/bjro/tzag003

**Published:** 2026-02-05

**Authors:** Po-Ting Lin, Chia-Yu Lin, Hsien-Tzu Liu, Jia-An Hong, Chih-Chien Li, Shan-Su Huang, Shu-Huei Shen

**Affiliations:** Department of Radiology, Taipei Veterans General Hospital, Taipei City 112, Taiwan; School of Medicine, National Yang Ming Chiao Tung University, Taipei City 112, Taiwan; Department of Radiology, Taipei Veterans General Hospital, Taipei City 112, Taiwan; School of Medicine, National Yang Ming Chiao Tung University, Taipei City 112, Taiwan; Department of Radiology, Taipei Veterans General Hospital, Taipei City 112, Taiwan; School of Medicine, National Yang Ming Chiao Tung University, Taipei City 112, Taiwan; Department of Radiology, Taipei Veterans General Hospital, Taipei City 112, Taiwan; School of Medicine, National Yang Ming Chiao Tung University, Taipei City 112, Taiwan; Department of Radiology, Taipei Veterans General Hospital, Taipei City 112, Taiwan; School of Medicine, National Yang Ming Chiao Tung University, Taipei City 112, Taiwan; Department of Radiology, Taipei Veterans General Hospital, Taipei City 112, Taiwan; Department of Medical Imaging and Radiological Technology, Yuanpei University of Medical Technology, Hsinchu City 300, Taiwan; Department of Radiology, Taipei Veterans General Hospital, Taipei City 112, Taiwan; School of Medicine, National Yang Ming Chiao Tung University, Taipei City 112, Taiwan

**Keywords:** ureteropelvic junction obstruction, computed tomography urography, aberrant renal vessels

## Abstract

**Objectives:**

To evaluate the diagnostic value of triple-bolus computed tomography urography (TB-CTU) for ureteropelvic junction obstruction (UPJO) in comparison with split-bolus CTU (SB-CTU).

**Methods:**

In this single-centre retrospective study, patients under clinical suspicion of UPJO referred from the urology clinic for CTU examination from January 1, 2017 to January 31, 2022, were included. CTU examinations were performed with SB or TB protocols. The images were reviewed by 2 radiologists for assessment of arterial and venous renal pelvis enhancement and arteriovenous differentiation. Interobserver agreement on arteriovenous differentiation was calculated.

**Results:**

A total of 23 TB-CTU and 70 SB-CTU examinations were included. The Hounsfield unit (HU) values for the renal artery, renal vein, and upper urinary tract were all significantly higher in the TB group. The proportion of high enhancement of arteries and veins was also significantly higher in the TB group (*P *< .001). Both radiologists evaluated TB-CTU as providing greater arteriovenous differentiation with strong interobserver agreement (κ = 0.77).

**Conclusions:**

TB-CTU exhibited superior arteriovenous differentiation in comparison with SB-CTU, with an acceptable radiation dose.

**Advances in knowledge:**

For patients with suspicion of UPJO, TB-CTU may be the imaging modality of choice for evaluating anatomical structures for further management.

## Introduction

Ureteropelvic junction obstruction (UPJO) refers to obstruction of urine flow at the junction of the renal pelvis and proximal ureter. It is a common cause of hydronephrosis in young patients and antenatal hydronephrosis.^1–4^ Men are affected by UPJO more commonly than are women. UPJO is more frequently identified on the left side.^5^

The pathophysiology of UPJO may be either congenital or acquired, with congenital UPJO being more common. Intrinsic narrowing is the most common cause of congenital UPJO, followed by compression by aberrant crossing vessels.^6–8^ The presence of aberrant vessels may influence surgical planning. Therefore, identifying the aetiology of UPJO is critical for its subsequent management.^7^^,^^9^^,^^10^

Computed tomography (CT) is an imaging modality commonly used to determine the aetiology of UPJO. Several protocols for urinary tract surveys have been introduced, including single-bolus triple-phase, split-bolus (SB) biphasic, and triple-bolus (TB) biphasic protocols.^11–14^ Single-bolus triple-phase CT urography (CTU) includes the image acquisition of the unenhanced, corticomedullary, nephrographic, and excretory phases. This multiphase protocol provides detailed data on the enhancement characteristics of renal lesions. However, the radiation dose caused by multiple image acquisitions is a problem.^12^^,^^13^

For SB-CTU, images of the unenhanced and excretory phases are acquired. The collecting systems are well enhanced as a result of a 15-min interval between the first and second boluses. The radiation dose may be lower in comparison with that in the single-bolus triple-phase protocol.^13^ However, the vascular structures and enhancement pattern of lesions may be poorly evaluated because only unenhanced and excretory phase images are acquired.^12^^,^^13^

Triple-bolus CTU (TB-CTU) has been introduced to display the enhancement phase with an acceptable radiation dose. TB-CTU is a dose-efficient protocol that effectively displays vascular enhancement phases.^11^

However, previous studies of TB-CTU have recruited patients with mixed symptoms, including haematuria, ureteronephrolithiasis, and urinary tract malignancy.^11^^,^^15^ Its value for the evaluation of UPJO remains unclear.

This study retrospectively evaluated the diagnostic value of TB-CTU for UPJO in comparison with SB-CTU.

## Methods

### Study population and design

This was a single-centre retrospective study. Patients under clinical suspicion of UPJO were referred from the urology clinic for CTU examination. This study included CTU performed under SB and TB protocols from January 1, 2017, to January 31, 2022. Patients with poor renal function (serum creatinine >2.0 mg/dL), age under 18 years old, and those with a history of malignancy were excluded.

The TB protocol was introduced in 2020 following the upgrade to a MEDRAD Stellant contrast media injection system (Bayer, Leverkusen, Germany); prior to this, only SB-CTU was utilized for evaluating suspected UPJO.

### Scanning protocol

**Figure 1.**
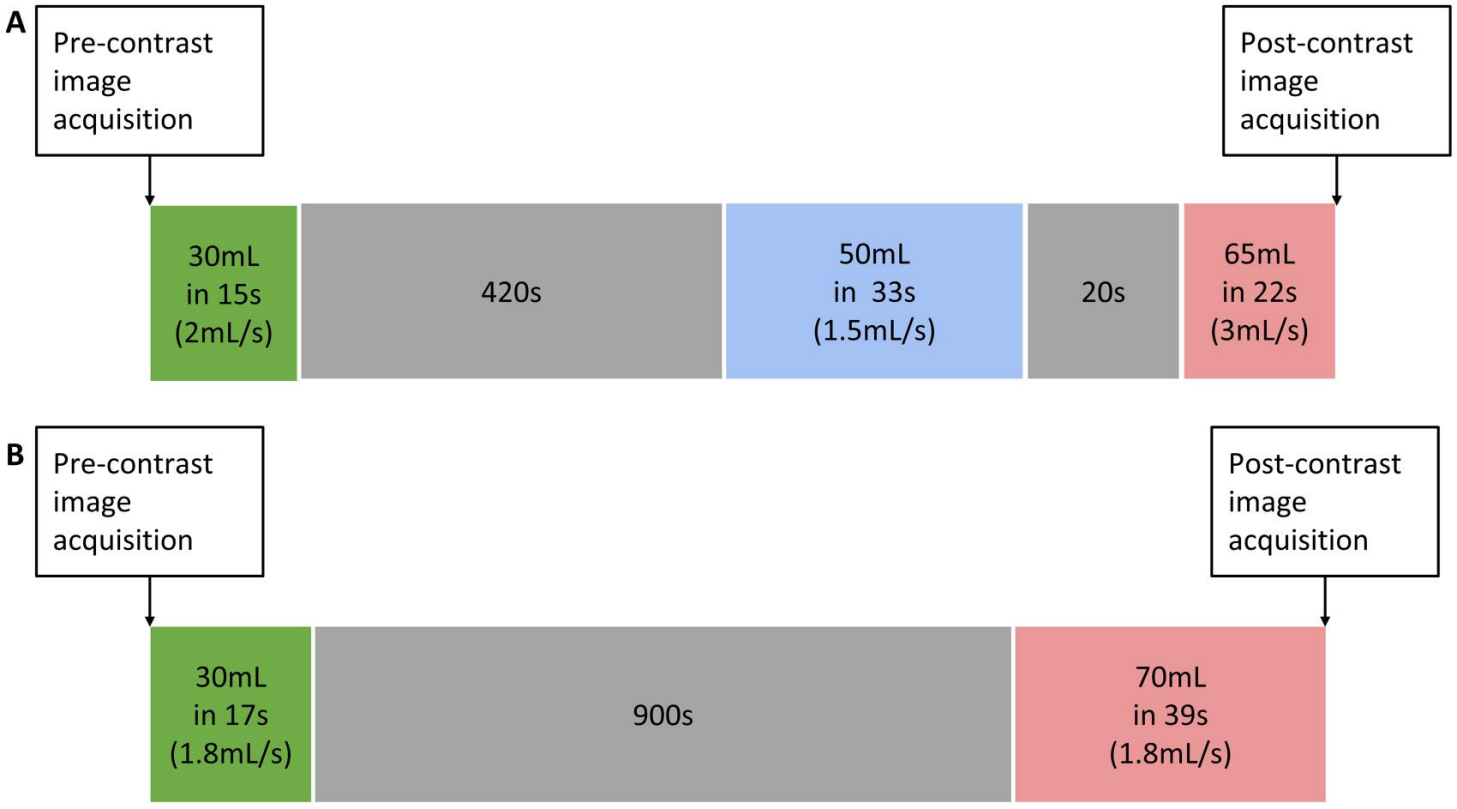
Schematic of computed tomography urography scanning technique of (A) triple-bolus and (B) split-bolus protocols.

Both SB-CTU and TB-CTU consisted of the acquisition of 2 scans: unenhanced and contrast-enhanced images. Unenhanced images were acquired before contrast injection, with a scanning range from the upper pole of the bilateral kidneys to the pubic symphysis in both protocols. A schematic of the CTU scanning technique for both protocols is presented in Figure 1.

For TB-CTU, the first bolus, 30 mL of contrast material was injected after the acquisition of unenhanced images, with an injection rate of 2 mL/s. After 420 s, 50 mL of contrast material was injected as the second bolus at an injection rate of 1.5 mL/s. The third bolus was injected 20 s after the second bolus, and 65 mL of contrast material at 3 mL/s was injected as the third bolus (Figure 1A). Contrast-enhanced images were acquired after the third bolus, with a scanning range of the diaphragm to the pubic symphysis.^11^ All TB-CTU examinations were performed using a single multislice CT scanner (Somatom Definition Flash, Siemens, Erlangen, Germany) and a MEDRAD Stellant contrast media injection system.

For SB-CTU, 30 mL of contrast material (iohexol, Omnipaque 350 mg iodine/mL, GE Healthcare; iopamidol, Iopamiro 370 mg iodine/mL, Bracco Diagnostics, Milan, Italy) was injected after the acquisition of unenhanced images. After 15 minutes, another 70 mL of contrast material was injected as the second bolus (Figure 1B). The injection rate of both boluses was 1.8 mL/s. Contrast-enhanced images were acquired after the second bolus injection from the diaphragm to the pubic symphysis.^12^^,^^13^ SB-CTU was performed using different multislice CT scanners (Aquilion Prime, Toshiba, Tochigi, Japan; Aquilion, Toshiba, Tochigi, Japan).

### Enhancement of anatomical structures

**Figure 2.**
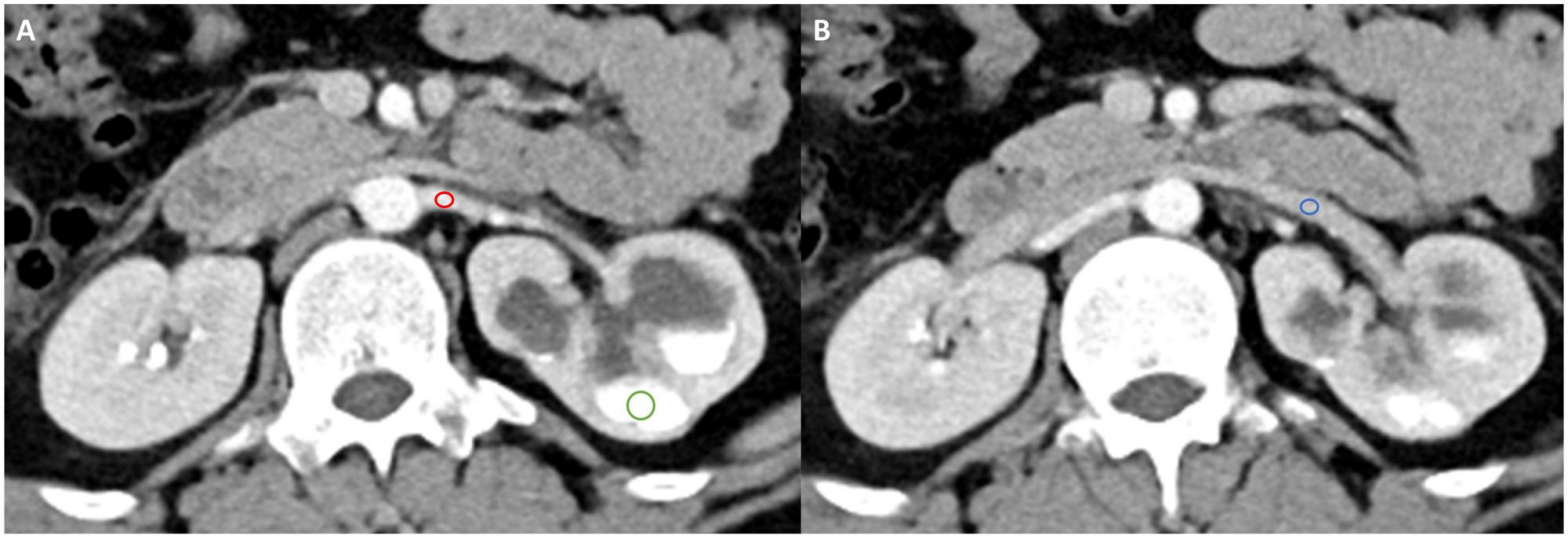
A 24-year-old male patient with clinically suspicious UPJO undergoes TB-CTU. The region of interest (ROI) is designated on the post contrast axial images for the calculation of Hounsfield units (HU). Assessment of enhancement in the renal artery, renal vein, and renal pelvis is conducted on the side where obstruction is suspected. The red circle in (A) denotes the ROI selected to evaluate the HU of the left main renal artery, while the blue circle in (B) designates the ROI for the left main renal vein. Emphasis on the upper urinary tract focuses on evaluating the enhancement in the renal pelvis. The green circle in (A) is chosen as the ROI for assessing the enhancement of the renal pelvis, targeting the most enhanced segment.

**Figure 3.**
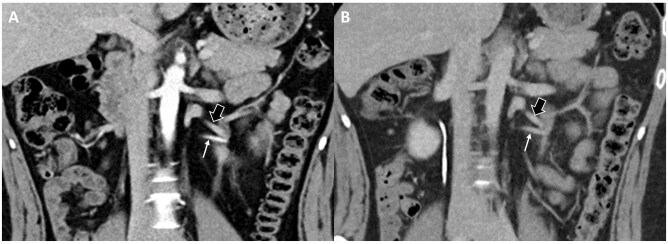
The same 24-year-old male patient in Figure 2 receives both TB-CTU (A) and SB-CTU (B) with an interval of 16 months. (A) On TB-CTU images, left aberrant renal artery (thin arrow in A and B) is well enhanced (score 3) and left aberrant renal vein (thick arrow in A and B) is diagnostically sufficiently opacified (score 2). Arteries and veins can be effectively differentiated, suggesting high AVD. (B) On SB-CTU images, both artery and vein are diagnostically enhanced (score 2) with similar density, indicating low AVD.

The images were reviewed by 2 radiologists: C.Y. Lin, with 4 years of experience, and P.T. Lin, with 3 years of experience in genitourinary radiology. The average Hounsfield unit (HU) values of the renal artery, renal vein, and upper urinary tract were evaluated in the postcontrast images by placing the region of interest (ROI) on the targeted structure on the picture archiving and communication system station. Assessment of enhancement in the renal artery, renal vein, and renal pelvis was conducted on the side where obstruction was suspected. Figure 2 illustrates the detailed evaluation of HU. The quality of renal vascular enhancement was evaluated by assigning a score for the artery and vein as follows: 3 indicated high opacification; 2 indicated diagnostically sufficient; and 1 indicated low or incomplete enhancement^11^ (Figure 3). The quality of upper urinary tract opacification was scored as follows: 3 indicated complete opacification; 2 indicated partial opacification (>50% of renal pelvis); and 1 indicated opacification <50% of renal pelvis.

Arteriovenous differentiation (AVD) was evaluated for both groups. If the artery and vein could be well differentiated in a single image, AVD was classified as being high. If the densities of the artery and vein were similar and could not be differentiated, AVD was classified as being low (Figure 3). The images were evaluated by the same 2 radiologists, and interobserver agreement was calculated.

For patients who underwent surgical intervention, operative findings were correlated with preoperative CTU images.

### Radiation dose

The effective radiation in both phases of the protocols was calculated according as the dose length product multiplied by a conversion factor of 0.015 according to The Measurement, Reporting, and Management of Radiation dose in CT, published by the American Association of Physicists in Medicine in 2008.^16^

**Figure 4.**
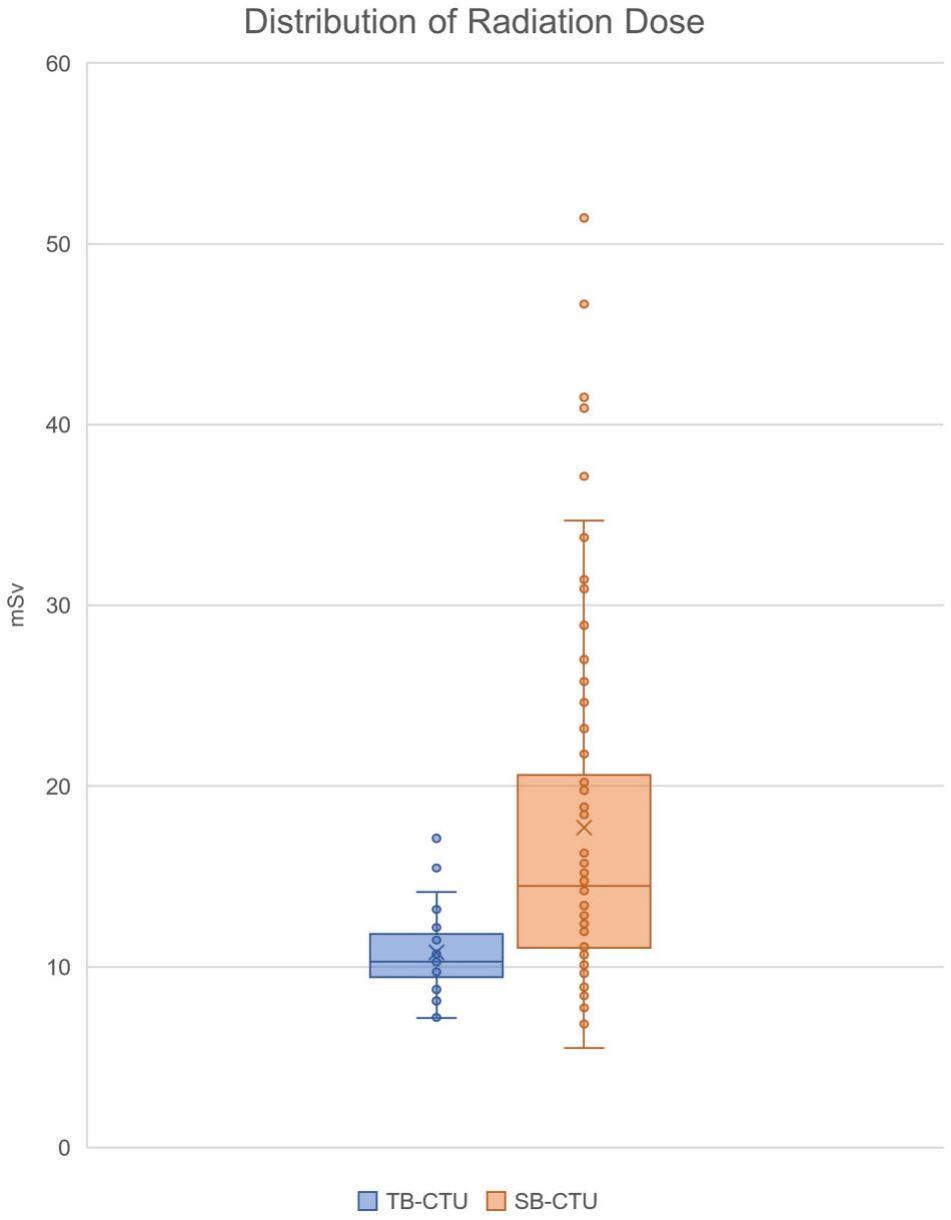
The box plot demonstrating the distribution of the radiation dose in both groups.

The effective dose was not calculated to estimate the radiation dose because the radiated regions were not uniform among patients. The distribution of radiation doses for both groups is presented in Figure 4.

### Statistical analysis

SSPS Statistics (version 25) was used. Patient characteristics that were continuous variables (ie, patient age, HUs, and radiation dose) were summarized as means and ranges. Those that were categorical variables (ie, sex, enhancement scale) were summarized as counts and percentages.

Interobserver agreement for AVD was calculated using the κ statistic (κ > 0.75 indicated high agreement; κ = 0.40-0.75 indicated medium agreement; and κ < 0.40 indicated low agreement).

## Results

### Patient characteristics

In total, 61 patients (93 examinations) were recruited under the clinical suspicion of UPJO and received 23 TB-CTU and 70 SB-CTU examinations. During the inclusion period, 16 patients only received TB-CTU, and 35 patients only received SB-CTU. Five patients received both TB- and SB-CTU. Of these 5 patients, 1 received 1 TB-CTU and 3 SB-CTU examinations, and the other 4 patients received 1 TB-CTU and 1 SB-CTU examination.

Patient characteristics are summarized in Table 1. Age and the mean creatinine level did not differ significantly between the 2 groups, but the TB-CTU group had a higher percentage of women (*P *= .01).

**Table 1.** Patient characteristics.

	Triple-bolus CTU	Split-bolus CTU	
No. of patients	21	40	
No. of examinations	23	70	
Mean age	39.8 ± 17.8	38.1 ± 15.5	*P* = .31
No. (percentage) of women	15 (65.2)	24 (34.2)	*P* = .01
Mean creatinine level (mg/dL)	0.78 ± 0.19	0.89 ± 0.19	*P* = .49
No. (percentage) of examinations with hydronephrosis	21 (91.3)	70 (100)	*P* = .06
No. (percentage) of examinations with aberrant artery	11 (47.8)	7 (10.0)	*P* < .001

Abbreviation: CTU = computed tomography urography.

### Detection rate of hydronephrosis and aberrant vessels

In the TB-CTU group, hydronephrosis was diagnosed in 21 (91.3%) examinations, with 13 cases (56.5%) on the left side, 7 cases (30.4%) on the right side, and 1 case (4.3%) on both sides. Parapelvic cyst was diagnosed in the remaining 2 examinations (8.7%). In the SB-CTU group, hydronephrosis was diagnosed in all 70 examinations with 52 cases (74%) on the left side and 18 (26%) on the right side. The percentage of hydronephrosis did not differ significantly between the groups (*P *= .06).

Aberrant renal arteries were identified in 11 of the 23 examinations (47.8%) in the TB-CTU group and in 7 of the 70 examinations (10.0%) in the SB-CTU group. The percentage of aberrant artery presence was significantly higher in the TB-CTU group than in the SB-CTU group (*P *< .001).

### Anatomical structure enhancement

**Figure 5.**
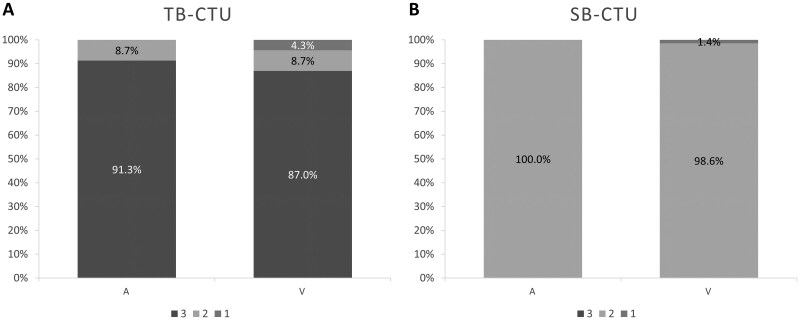
Bar graph demonstrating proportion of artery and vein enhancement scores for (A) TB-CTU and (B) SB-CTU (3, high opacification; 2, diagnostically sufficient; 1, low or incomplete enhancement).

The proportions of the enhancement scores of arteries and veins under both protocols are displayed in Figure 5. Most arteries exhibited high enhancement (91.3% for arteries and 87.0% for veins) in the TB-CTU group. In the SB-CTU group, the enhancement of most of the vessels was classified as being diagnostically sufficient, and none of the arteries was classified as having high enhancement. The proportion of high enhancement of both arteries and veins was significantly higher in the TB-CTU group (*P *< .001, Figure 5). The HU values of the renal artery and renal vein were significantly higher in the TB-CTU group than in the SB-CTU group (*P *< .001 for both arteries and veins). The detailed statistics are demonstrated in Table 2.

**Table 2.** HUs of renal artery and renal vein.

HUs	Triple-bolus CTU (*n* = 23)	Split-bolus CTU (*n* = 70)	
Artery (A)	275.2 ± 67.7	145.0 ± 27.8	*P* < .001
Vein (V)	166.9 ± 56.0	144.2 ± 28.1	*P* < .001

Abbreviations: CTU = computed tomography urography; HU = Hounsfield units.

**Figure 6.**
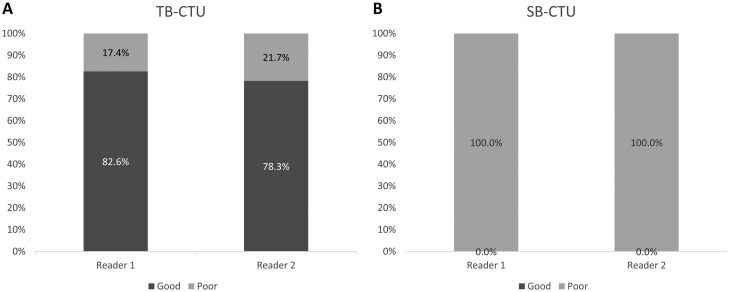
Twenty-three examinations were selected from each group, respectively. Bar graph displays proportions of high and low AVD evaluated by 2 radiologists for (A) TB-CTU and (B) SB-CTU. Interobserver agreement was strong (κ = 0.77).

Twenty-three examinations were selected from each group for double-checking of AVD by the 2 in-charge radiologists. In this evaluation, the percentage was 100% (23/23) and 32.9% (23/70) in the TB-CTU and SB-CTU groups, respectively. Both radiologists rated the TB-CTU images as exhibiting higher AVD, with strong interobserver agreement (κ = 0.77) (Figure 6).

In both groups, approximately half of the upper urinary tract images exhibited less than 50% opacification because of urinary tract obstruction (Table 3). The quality of upper urinary tract opacification did not differ significantly between the 2 groups (*P *= .25).

**Table 3.** Quality of upper urinary tract opacification.

No. of examinations	Triple-bolus CTU (*n* = 23)	Split-bolus CTU (*n* = 70)
Complete opacification	7 (30.5%)	25 (35.7%)
Partial opacification (>50%)	5 (21.7%)	13 (18.6%)
Opacification <50%	11 (47.8%)	32 (45.7%)

Abbreviation: CTU = computed tomography urography.

### Correlation with operative findings

**Figure 7.**
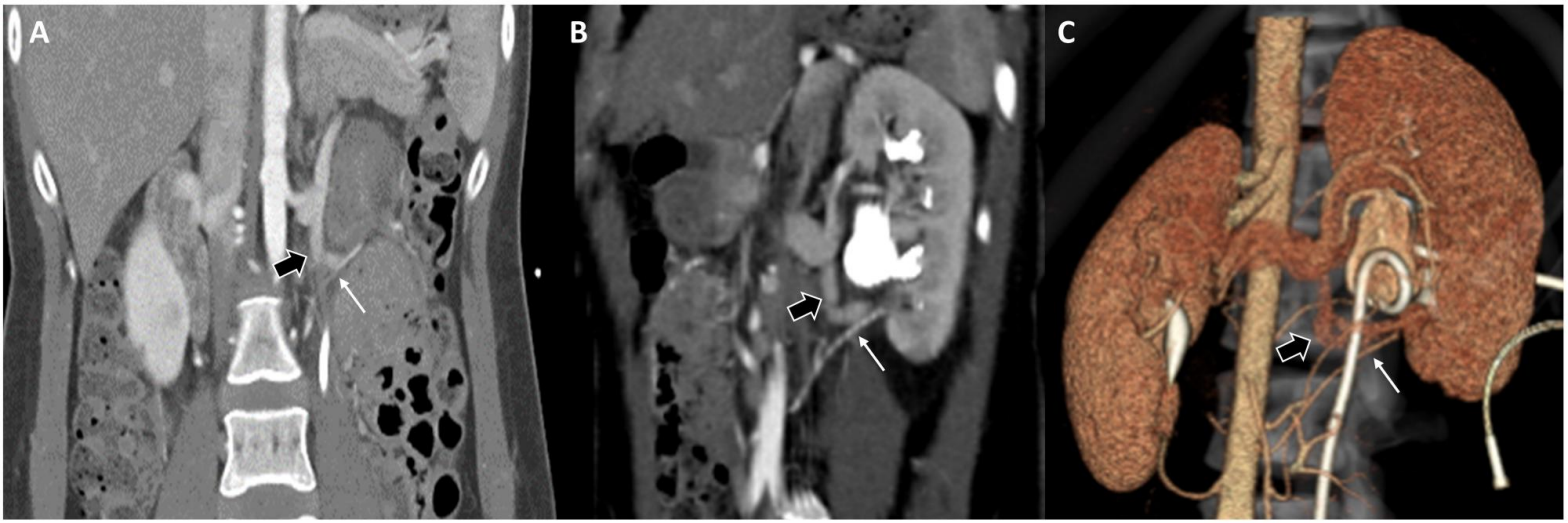
A 29-year-old female patient with intermittent left flank pain received TB-CTU under impression of UPJO. Coronal reformatted image (A) and oblique coronal reformatted image (B) demonstrate aberrant renal artery (thin arrow in A, B, and C) crossing left ureteropelvic junction. (C) Reconstructed image with 3-dimensional volume rendering technique presents relationship of aberrant renal artery and extrahilar confluence of left renal vein (thick arrows in A, B, and C) and left ureter (with ureteral stent implantation). Imaging findings were confirmed by surgery.

Nine patients in the TB-CTU group (42.6%) and 27 patients in the SB-CTU group (67.5%) received further surgical intervention for UPJO. Aberrant vessels were identified during surgery in 2 of the 9 patients in the TB-CTU group and in 3 of the 27 patients in the SB-CTU group, which was consistent with presurgical diagnoses (Figure 7). As surgical intervention, pyeloplasty was performed, and the other patients were treated conservatively by relieving obstructions using ureteral stenting or observation.

### Radiation dose

The radiation dose in the TB-CTU group was significantly lower than that in the SB-CTU group (10.8 ± 2.4 vs 17.7 ± 9.9 mSv; *P *< .001), and the distribution of radiation doses for both groups is presented in Figure 4. This may be because the 19 SB-CTU examinations involving radiation doses of over 20 mSv were performed using a CT scanner (Aquilion, Toshiba, Tochigi, Japan) manufactured in 2006. The older versions of the software and algorithms used by this scanner differed from those of other scanners, which led to a higher average radiation dose when performing the same protocol. After excluding CTU examinations performed using this scanner, the radiation dose did not differ significantly between the TB-CTU and the SB-CTU groups (10.8 ± 2.4 vs 12.6 ± 3.3 mSv; *P *= .05).

## Discussion

**Figure 8.**
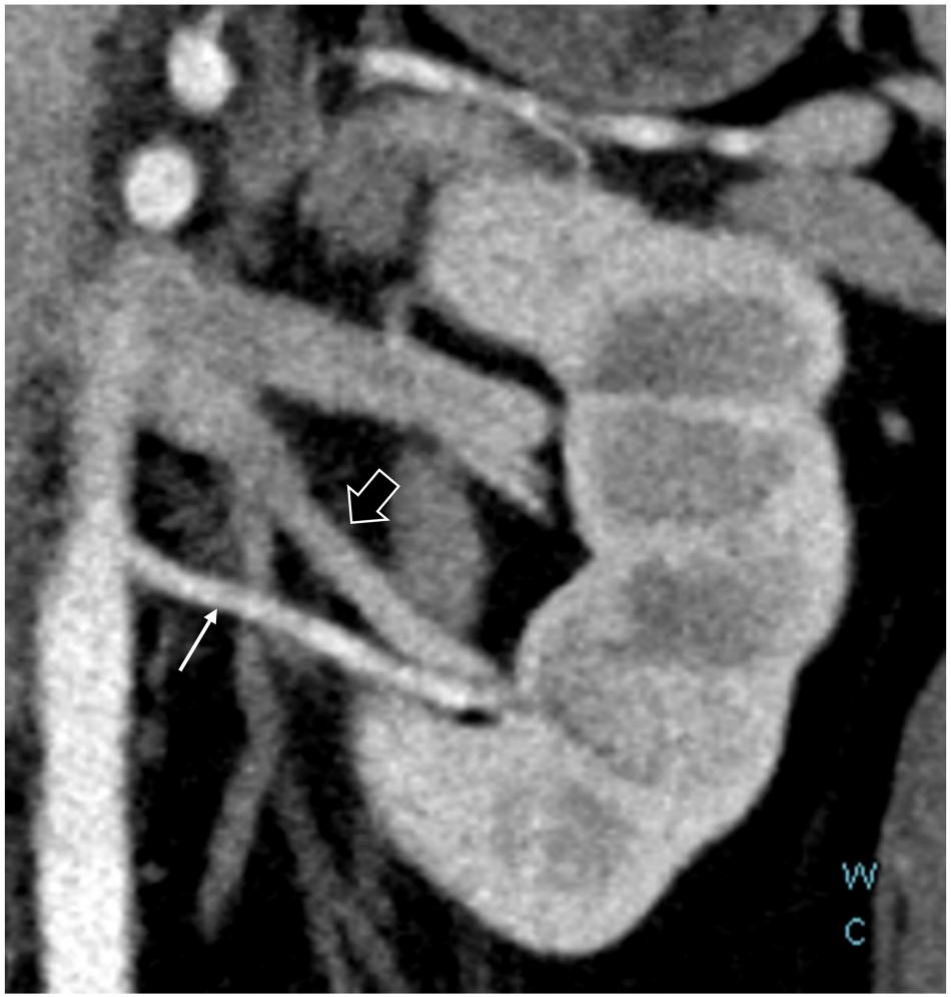
A 24-year-old male patient with clinical suspicious UPJO. Oblique coronal reformation imaging of TB-CTU examination displays left aberrant renal artery (thin arrow) and extrahilar confluence of renal veins (thick arrow), with vein draining inferior portion of left kidney confluent with gonadal vein.

**Figure 9.**
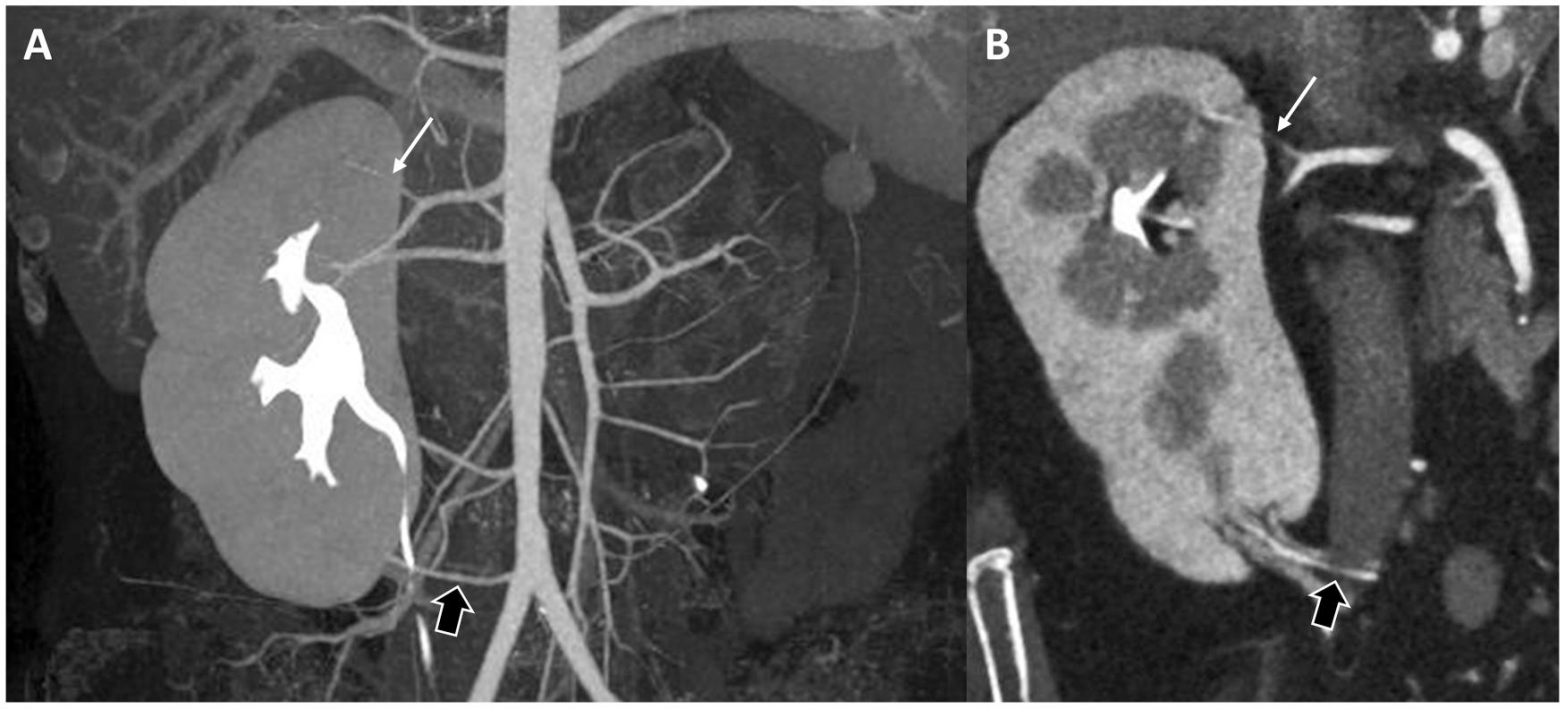
A 27-year-old female patient with history of Turner syndrome receives TB-CTU for evaluating left UPJO with atrophic left kidney. (A) Maximum intensity projection image and (B) oblique coronal reformation image depict 3 right renal arteries with capsular artery supplying upper pole (thin arrow) and one polar artery from right common iliac artery supplying lower pole (thick arrow). Relationship of arteries, veins, and ureter can be demonstrated in a single scan.

The results of this study demonstrated that TB-CTU protocols can demonstrate the relationship between the arteries, veins, and urinary tract in a single scan (Figures 8 and 9). The radiation dose involved is considerably lower than that in traditional multiphasic CTU protocols. As compared with SB-CTU, the TB-CTU protocol has higher volume and higher injection rate and consequently, increased iodine delivery rate. Therefore, both artery and vein showed higher attenuation in TB-CTU as compared with in SB-CTU, as demonstrated in Table 2. Furthermore, by dividing the contrast bolus into 3 parts, the TB-CTU protocols may effectively increases the AVD and displayed the arterial structure more clearly. It is crucial to discern variations in renal arteries and veins prior to surgery. While certain variants may be detectable in SB-CTU, discrepancies in arterial and venous variations may exist within the same individual, as illustrated in Figures 3 and 8. With high AVD and acceptable radiation dose, TB-CTU is considered suitable for preoperative evaluation and follow-up in young patients.

CT is a non-invasive imaging modality used to provide detailed anatomical information for the evaluation of UPJO.^17–19^ Although concerns exist regarding the radiation dose and the side effects of contrast media injection, time efficiency and high spatial resolution are the advantages of CT over other imaging modalities such as ultrasound and magnetic resonance imaging (MRI).^5^ Different scanning protocols have been applied for different urinary tract pathologies.^11–14^ CTU has been proposed as the optimal scanning strategy for evaluating the urinary tract, and it has been widely applied for the evaluation of kidney stones and urinary tract malignancy. The primary purpose of CTU in patients with suspected urinary tract malignancy is to detect intraluminal lesions within the collecting system. However, CTU protocols designed for evaluating urinary tract malignancy may be suboptimal for assessing arterial anatomy. For patients with UPJO, detecting the presence of aberrant vessels is crucial for presurgery evaluation. The presence of crossing vessels over the UPJ may be the direct cause of UPJO. Even if it is not, it may be associated with the risk of ischaemia or haemorrhage during pyeloplasty.^10^^,^^18^^,^^19^ Therefore, identifying the presence of these vessels is critical for surgical planning.^5^^,^^7^^,^^18–20^

CT angiography with 3-dimensional reconstruction has been proposed as an accurate imaging modality for detecting aberrant vessels for preoperative evaluation in patients with UPJO.^19^^,^^21^^,^^22^ Farrés et al. reported that macroscopic surgical findings agreed with CT findings in all 20 patients (100%). Khaira et al. compared the findings of single-bolus contrast-enhanced arterial and excretory phase CT with findings from laparoscopic or open pyeloplasty. The detection of crossing vessels did not differ, and specificity was 100%.^23^ Because patients with UPJO are relatively young and usually require repeat follow-up imaging surveys, the radiation dose should be considered according to the as low as reasonably achievable principle.^3^^,^^15^

Other imaging tools can be used to evaluate the urinary tract. Ultrasound is a non-invasive, cost-effective, and easily accessible imaging modality for surveying urinary tract obstruction. Dilatation of the proximal ureter and renal pelvis without a distended bladder indicates obstruction of the ureter.^3^^,^^5^^,^^24^ MRI can be used to accurately confirm the anatomy and identify the point of obstruction. The disadvantages of MRI include higher costs, greater time consumption, and the necessity for patients to hold their breath. General anaesthesia or sedation may be required for paediatric patients.^3^^,^^7^^,^^24^ Diuretic renal scanning (or renal scintigraphy) is a nuclear medicine examination for detecting urinary tract obstruction. The most commonly used radioisotope is technetium-99m-mercaptoacetyltriglycine. Diuretic renal scanning is used to measure the drainage time from the renal pelvis and to assess the percentage of relative function of each kidney. The washout measurement of the radioisotope is correlated with the degree of obstruction. A half-life of more than 20 min indicates delayed transient washout, which indicates significant obstruction. A difference of more than 10% in split renal function identified using diuretic renal scanning also indicates obstruction.^24–26^ However, none of the aforementioned imaging techniques provide detailed anatomical information as provided by CT.

This study has several limitations. First, selection bias may exist between the 2 groups because the percentage of aberrant vessels was significantly higher in the TB-CTU group. Additionally, the introduction of the TB protocol in 2020 following the upgrade of the injector resulted in a difference in case numbers between the 2 groups. Second, the volume of contrast medium injected varied between the groups, potentially leading to differences in enhancement levels and HU measurements during image evaluation. Third, the diagnosis of aberrant vessels was based on imaging studies, and not all patients received surgical confirmation. Fourth, SB-CTU examinations were not undertaken using the same CT scanner, whereas the TB-CTU examinations were performed using the same CT scanner.

In conclusion, TB-CTU protocol effectively displays the vascular and urinary tract anatomy of patients with UPJO with an acceptable radiation dose, and it may provide sufficient information for further management and surgical intervention.
